# Fluorescence Molecule Counting for Single-Molecule Studies in Crowded Environment of Living Cells without and with Broken Ergodicity

**DOI:** 10.2174/138920111795470949

**Published:** 2011-05

**Authors:** Zeno Földes-Papp, Gerd Baumann

**Affiliations:** 1 Medical University of Graz, Riesstrasse 58a/5, A-8047 Graz, Austria; 2 Mathematics Department, German University in Cairo

**Keywords:** Anomalous motion, broken ergodicity, continuous time random walks (CTRW), continuous time random walks (CTRW) on fractal supports, Limited Continuous Time Random Walks (LCTRW) on fractal supports, molecular crowding, ergodicity, FCS, FCCS, fluorescence fluctuation microscopy, heterogeneity, living cells, complex body fluids like blood and its components, interpretation of subdiffusive measurements, meaningful time for studying just one single molecule, physical model of crowding, physical model of temporal heterogeneity, random walks on fractal supports, resolution limits of measured diffusion times for two components, temporal autocorrelation, temporal two-color crosscorrelation, fluorescence imaging, time dependence of apparent diffusion coefficients.

## Abstract

We present a new approach to distinguish between non-ergodic and ergodic behavior. Performing ensemble averaging in a subpopulation of individual molecules leads to a mean value that can be similar to the mean value obtained in an ergodic system. The averaging is carried out by minimizing the variation between the sum of the temporal averaged mean square deviation of the simulated data with respect to the logarithmic scaling behavior of the subpopulation. For this reason, we first introduce a kind of Continuous Time Random Walks (CTRW), which we call Limited Continuous Time Random Walks (LCTRW) on fractal support. The random waiting time distributions are sampled at points which fulfill the condition *N* < 1, where *N* is the Poisson probability of finding a single molecule in the femtoliter-sized observation volume ΔV at the single-molecule level. Given a subpopulation of different single molecules of the same kind, the ratio *T/ T_m_* between the measurement time *T* and the meaningful time *T_m_*, which is the time for observing just one and the same single molecule, is the experimentally accessible quantity that allows to compare different molecule numbers in the subpopulation. In addition, the mean square displacement traveled by the molecule during the time t is determined by an upper limit of the geometric dimension of the living cell or its nucleus.

## 1 INTRODUCTION

Measurements of single-molecule fluctuations have a long history as an essential tool for studying diffusive and kinetic properties in confocal microscopy and spectroscopy [1]. For example, in dilute solutions fluctuations in the fluorescence intensity are caused by fluctuations in the local concentration of fluorescent molecules. Fluorescence correlation spectroscopy (FCS) measures the fluctuations of the collected fluorescence intensity from the time average value of the fluorescent species emitting photons at a certain value of wavelengths (emission peak maximum) in a laser-illuminated observation volume ∆V. Moving on from time-averaged ensemble measurements to studies on time-averaged single enzyme molecules immobilized on cover slips allows for detection of dynamic disorder, i.e. time-dependent fluctuations in the single-enzyme turnover [2-4].

Enzymes and proteins in their natural environment of living cells or body fluids like blood and its components interact with a network of numerous neighboring proteins, and their activity depends on the local environment and their role in the catalytic cycle [5]. As a consequence, the observed dynamic properties are related to the time since the system is switched on (also called waiting time). This very slow phenomenon is known as aging. One of the new developments concerns the breakdown of the fluctuation-dissipation theorem [6] in subpopulations of single molecules. Theoretical arguments and some experimental data show that slow approaches to equilibrium influence fluctuations whose time scale is much shorter than the time for which the ensemble of single molecules shows non-stationary increments and gives rise to ergodicity breaking and aging [7, 8]. 

In recent years, evidence has accumulated for anomalous subdiffusive motion of molecules in various eukaryotic systems [7, 9, 10]. The eukaryotic cytoplasm contains different organelles, an elaborated cytoskeleton, and various mechanisms for active transport of molecules in the cell and the cellular compartments like nucleus. Values of $\tilde{\gamma }$ involve subdiffusion with $\tilde{\gamma }<1$, normal diffusion with $\tilde{\gamma }=1$ as well as superdiffusive motion with $\tilde{\gamma }>1$ suggesting the occurrence of active transport. In biological cells, the motion of proteins can be hindered either by molecular crowding or/and by chemical binding [7]. The important distinction being made by us is between space (structure)-dependent and time (rate)-dependent sources for anomalous diffusion [11]. The molecules are not immobilized on a solid support (solid phase) and they are not hydrodynamically or electrokinetically focused.

In this original paper, we specifically address the question of discriminating between spatial and temporal randomness that both lead to anomalous, subdiffusive motion of single molecules in living cells and their compartments like the nucleus or in body fluids like blood and its components. We quantitatively describe the network of molecular interactions of single molecules by the product $\tilde{\gamma }=\alpha \cdot \gamma \cdot \alpha$ accounts for the molecular crowding and γ for the temporal heterogeneity. The paramete γ controls the dynamics of the interaction network. In our computational model, *γ* depends on the waiting time distribution of the single biomacromolecule to be trapped in interactions with its neighboring ligands or reaction partner(s). Unbroken and broken ergodicity enter the problem by taking averages in the population of single molecules. A physical process is ergodic if the ensemble average over many single molecule trajectories coincides the time average, i.e. a moving average over a single molecule trajectory of time length *T*. Broken ergodicity means that both averages are different. In our case, using anomalous diffusion without broken ergodicity and anomalous diffusion with broken ergodicity would have the advantage of simulating experimentally accessible parameters like *on-off* events, diffusion times and apparent diffusion coefficients, respectively, and temporal resolution limits of different single molecular species according to their mass differences [11]. As proven here for the first time, performing ensemble averaging in a sparse subpopulation of such individual molecules during measurement leads to a mean value that can be similar to the mean value obtained in an ergodic system. Thus, broken ergodicity and unbroken ergodicity are not anymore distinguishable. In living cells or body fluids like blood and its components, ensemble and temporal averaging are carried out without knowing whether the underlying molecular system behaves in ergodic or non-ergodic ways. Yet the theory predicts that each measurement can be related to an ergodic or a non-ergodic behavior unless one is able to show the single-molecule fingerprint of non-ergodicity.

## 2 THEORY

The essential ingredient of modeling the molecular crowding is the random walk of a molecule on fractal support that is taken as power law with a certain crowding exponent α [11]. Our choice was motivated by the presence of diffusive obstacles of many different sizes. These fractal supports have holes on every length scale due to their construction procedure. Therefore, the diffusive motion of the molecule on such structures is slowed down at time *t*. The resulting diffusive law becomes subdiffusive [12]

(1) $$\langle {\vec{r}}^{2}\left(t\right)\rangle =\Gamma_{\alpha }\cdot t^{\alpha }\propto t^{\alpha }\cdot$$

The mean square displacement (*MSD*) of the molecules $\langle {\vec{r}}^{2}\left(t\right)\rangle$ in *n*-dimensional space is anomalous and scales with the crowding exponent α (0 < α < 1) [11]. 

If there is anomalous diffusive motion of molecules in a living cell due to a trap mechanism, then there must be some biological event that turns on the interaction with the traps. There are many possibilities for such an event [13]. Changes in localization like entry of a DNA-binding regulatory protein into the nucleus or assembly of a functional enzymatic complex or conformational changes in the diffusing species or binding of a ligand to a receptor or (de)phosphorylation can occur. We have first proposed an analysis concept in which diffusive motion is inherently linked to cellular metabolism [11]. Our analysis concept differs from refs. [2-5, 7-10, 12, 13] by its advantage to directly count the number of molecules in the femtoliter-sized observation/detection volume ΔV in the dilute solution or living cell. Hence, we refer to it as fluorescence molecule counting for single-molecule studies in the crowded environment and living cells. The determination of the molecule number per ΔV is used to follow cell biological processes in time. One way that the single molecule can be probed in fluorescence molecule counting is by gathering the time dependent response as a time series measurement, e.g. a time-lapse measurement. In order to take account of temporal randomness of molecular interaction, i.e. temporal heterogeneity, during subdiffusive motion of a single molecule, we perform the random walk on the fractal support as a continuous time random walk (CTRW) [11]. The *MSD* traveled by the molecule during the time *t* is given by the law

(2) $$\langle {\vec{r}}^{2}\left(t\right)\rangle \propto t^{\alpha \cdot \gamma }=t^{\tilde{\gamma }}\cdot$$

The important feature of this law (Eqn. 2) is that the spatial and temporal coordinates are decoupled. α stands for the molecular crowding and γ for the temporal randomness of a trapping mechanism. We quantitatively take both cellular restraints into account by the product $\alpha \cdot \gamma =\tilde{\gamma }$. 

The molecule has to wait for a time *t* on each site of the fractal support before performing the next step. The waiting time is a random variable independently chosen at each new step according to a continuous distribution *ψ*(*t*). In our case [11], the inverse gamma distribution was used to generate the waiting time steps $\psi \left(t\right)=e^{-1/\left(t\right)}t^{-\left(1+\gamma \right)}/\Gamma \left(\gamma \right)\propto t^{-\left(1+\gamma \right)}\Gamma \left(\gamma \right)$, where Γ(γ) here is the gamma function. It is a well-known result that since the first moment is infinite the central limit theorem does not apply. The inverse gamma distribution was used because of the occurrence of heavy-tailed CTRW. Hence, it is very clear that the ergodicity is broken on all time scales *t* and we exactly simulated and predicted the behavior of a selfsame molecule in a crowded environment with temporal randomness [11, 14].

Since the experimental conditions to measure a selfsame molecule over an extended period of time, at which biology is taken place, in living cells and body fluids like blood and its components or even in dilute solutions are very restrictive [14-17], temporal disorder can be mimicked through waiting time distributions *ψ*(*t*) displaying long-time tails

(3) ψt∝t−1+γ with 0 <α,γ∼<1.

We need to perform the time average over a subpopulation of different single molecules of the same kind

(4) $${\langle {\langle {\vec{r}}^{2}\left(t\right)\rangle }_{T}\rangle }_{\mathrm{sub}-\mathrm{ens}}={\langle {\langle {\vec{r}}^{2}\left(t\right)\rangle }_{\mathrm{sub}-\mathrm{ens}}\rangle }_{T}={\langle {\langle n^{\tilde{\gamma }}\left(t\right)\rangle }_{\mathrm{sub}-\mathrm{ens}}\rangle }_{T}\cdot$$

*n* here represents the diffusive steps of the single molecule. The two averaging procedures in Eqn. (4) are interchangeable. Our experimental single-molecule regime given by Eqn. (4) [11] and in our papers [14-17] differs from averaging over the whole molecule ensemble suggested by Meroz, Sokolov, Klafter (2010) [7]. We perform averages in sparse subpopulations of single molecules, i.e. in sub-ensembles of single-molecules that are abbreviated by the subscript sub-ens and the shorter sub for sub-population, respectively.

In this article, we shall examine the time and spatial dependence of the heterogeneous exponents

(5) α⋅γ = γ∼ with 0<α,γ∼<1.

We present an approach on how to decide from a subset of single-molecule measurements how heterogeneous the studied system is in time. The single molecules are not immobilized on a solid support and they are not hydrodynamically or electrokinetically focused. We theoretically describe the network of molecular interactions in living cells or body fluids like blood and its components by the product $\tilde{\gamma }$. Thus, the important distinction is first made between space (structure)-dependent and time (rate)-dependent sources for anomalous diffusive motion. 

## 3 METHODS AND SIMULATION

Various experimental methods have been applied to large-scale studies of proteins and protein networks, including mass spectrometry, protein chips, and two-hybrid screening [18-24]. Proteome studies using autofluorescent fusion proteins have also been performed [25]. These methods yield only qualitative data. The suitability of fluorescence correlation spectroscopy for high-throughput data acquisition was shown [26]. There is a need for new techniques in order to quantify cellular protein networks. The first steps towards this goal include computational approaches and can experimentally be performed by focusing on selected pathways. However, quantitative studies of protein-protein interaction networks are still in their infancy. Quantitative data from these *in vitro* interaction studies do not fulfill the requirements for standardization of the measurement conditions and they are of non-physiological nature due to the cell-free approaches. Therefore, we theoretically predict the collective influence of a molecular interaction network on the behavior of single biomacromolecules in living cells or body fluids like blood and its components or in dilute solution. The influence of the molecular interaction network is quantitatively expressed by the heterogeneity parameter γ in Eqn. (5).

In Baumann and Földes-Papp 2010 [11], we have first established the most generally applicable method for data analysis of diffusive measurements in living cells or body fluids like blood and its components under crowded and heterogeneous conditions for two (dim = 2) or three (dim = 3) dimensions

(6) $$D_{\mathrm{app}}\left(t\right)=\frac{\Gamma_{{\tilde{\gamma }}_{\ell }}}{2\cdot \dim}\cdot t^{\tilde{\gamma }-1}$$

such that the *MSD* can then be written as

(7) $$\langle {\vec{r}}^{2}\left(t\right)\rangle =2\cdot \dim\cdot D_{\mathrm{app}}\left(t\right)\cdot t\cdot$$

Here, $\Gamma_{\tilde{\gamma }\ell }$ is a pre-factor with dimensions of length-squared per fractional time *t*. For the first time, specific examples of Eqs. (6) and (7) were theoretically analyzed in ref. [11]. Again, Eqs. (6) and (7) governing crowded *and* temporally heterogeneous motion of molecules at the many-molecule and single-molecule levels give a complete picture concerning this subject. The existence of different exponents in Eqs. (6) and (7) is an important property of the product $\tilde{\gamma }$ of randomness in cellular systems and justifies the following concept: We first noted in ref. [11] that there is a normalized auto- (and two-color cross)correlation *G*(*τ*) associated with Eqs. (6) and (7)

(8) Gτ = 1N⋅1+ττDγ∼−1⋅1+1S2ττDγ∼−dim−2/2+1,

where *τ* is the correlation time with $\lim_{\tau \to 0}=\frac{1}{N}+1$, *N* is the molecule number per femtoliter-sized observation volume ΔV at the many-molecule level or the Poisson probability of finding a single molecule in ∆V at the single-molecule level with *N* < 1 [14-17], *τ_D_* is the diffusion time that is a specified correlation time τ, and *s* here is a so-called structural factor that is defined, for instance, as $s=z_{0}/\omega_{x-y}$ with the half-length *z*_0_ and the radial waist *ω*_x-y_ of ΔV, dim = 3 for 3D measurements and dim = 2 for 2D measurements in membranes. Time traces that are recorded for the subpopulation of single molecules without interacting partner, e.g. without ligand, in the crowded environment of living cells and their cellular compartments, respectively, or body fluids like blood and its components yield $\tilde{\gamma }=\alpha$. The molecular crowding parameter α (0 < α < 1) can be measured in the absence of ligand(s) by means of Eq. (8) in fluorescence correlation spectroscopy. Knowing the molecular crowding parameter α for the cell type and cellular compartment type in the absence of ligand(s), the parameter of temporal heterogeneity γ can be extracted from the measurements in the presence of interacting partner(s), e.g. ligand or neighboring protein(s), for the same α with $0<\alpha ,\tilde{\gamma }<1.\gamma$ (Eqn. (5)) is not a simple fitting parameter. More specifically, one can inquire if spatial and temporal randomness in the single-molecule trajectories can supply additional information useful in discriminating between crowding and heterogeneous dynamic behavior of interactions with neighboring proteins or ligands in living cells or in body fluids like blood and its components.

If the molecular crowding is separated from temporal interaction rather than taking the usually non-separated form as a single dynamic exponent, then this view provides a straightforward explanation for the apparently different behavior of different classes of biomacromolecules like DNA, RNA, proteins in live cells *and* dextran molecules in solution ranging from 10 KDa to 2 MDa. So far, measurements in the literature only consider a single “dynamic” exponent, e.g. refs. [27-29, 9]. To our knowledge, experiments in living cells have never measured a value 0 < α < 1 solely due to molecular crowding; molecular interaction were always involved to get 0 < α < 1.

According to ref. [12], we obtain

(9) τDt = ωx−y24⋅m⋅Γγ∼ℓ/2⋅dim1γ∼ = ωx−y24⋅m⋅Dappt = γ∼⋅ωx−y24⋅m⋅Dinstt

*D_app_* is the measured or apparent diffusion coefficient, e.g. in fluorescence correlation spectroscopy. *m *specifies one- (*m *= 1) or two-photon (*m *= 2) excitation. With this clarification, it would become more feasible to unambiguously report mobility data in terms of either a time-dependent diffusion time *τ_D_* or as time-dependent diffusion coefficient [30-33]. We do not discuss this subject here and refer to ref. [11]. 

We have applied random walks on fractal supports without continuous waiting time distributions (so-called random walks on fractal structures, RWF) and with continuous waiting time distributions (so-called continuous time random walks, CTRW) [34, 35]. Here, we give a brief summary how the simulations were carried out. For a more detailed discussion, we refer to our papers [11] and [17]. We generate a random Brownian walk by randomly selecting steps in the three coordinate directions. The three coordinate directions are generated by a permutation of the vector **v**=(0,0,1) so that a set of orthogonal vectors ***S*** is generated. Mathematically this means we use the basic set of orthogonal unit vectors in a Cartesian coordinate system as the basis of our calculations. This set of permutated vectors is extended in all directions positive and negative by the following unification of basis sets. Introducing the random function *R_k_*, which selects the direction with equal probability randomly from our basis set S*, we create the Brownian track $B_{n}\left({\vec{r}}_{0}\vec{r}\right)$ by a sum of independent vectors. ${\vec{r}}_{0}$ is the origin of the track of *n*-steps represented as continuous function $B_{n}\left({\vec{r}}_{0}\vec{r}\right)$ for the end point$\vec{r}$. The corresponding generating function is $H\left(z,{\vec{r}}_{0},\vec{r}\right)=\sum_{{n=0}^{z^{n}}}^{\infty }B_{n}\left({\vec{r}}_{0},\vec{r}\right)$, which allows us to define the moments of the walk. This generation of a fractal is based on the renormalization of the whole structure and can be used efficiently to generate a fractal support on an infinite space. We perform random walks on these lattices. By the same method we generalized the Sierpinski gasket and the carpet to a different structure, if we not only delete one element in the generator but, instead, allow the deletion of more than one element. This, of course, results in a great variety of generalized Sierpinski patterns introducing a variation of the gasket and the carpet. In our examinations, we will restrict us to generalized Sierpinski carpets (GSC), which delete not more than half of the elements of the generator. We only supply the generator as input. The random walker is set on some site and it tests whether each site it arrives at is an allowed site, as it goes along. This kind of walk generation is known as the blind ant approach. The actual procedure is as follows: The walk can start at any site of the underlying virtual lattice. To check whether a site is accessible, the first step is to identify the iteration stage the point belongs to. For any *ν^3^* grid, a point having either an x^(*i*)^-coordinate (*i *= 1, 2, 3) between *ν*^*k*-1^ and *ν^k^* belongs to the *k*th iteration stage of the fractal. In the *k*th-stage coarse-grained pattern with units of size *ν*^*k*-1^, it is checked whether the block containing the site matches an accessible site on the given generator. If found accessible, the corresponding point in the next lower stage, i.e.(*k*-1), is ascertained. In this way, the point is successively scaled down until it reaches the first stage. In general, in the *k*th stage, the equivalent coordinates $\left(x_{k}^{\left(1\right)},x_{k}^{\left(2\right)},x_{k}^{\left(3\right)}\right)$ are given by the integer parts of $x_{k}^{\left(i\right)}/\nu^{k-1}$ with *i* = 1,2,3. If $\left(x_{k}^{\left(1\right)},x_{k}^{\left(2\right)},x_{k}^{\left(3\right)}\right)$ matches an allowed site, the coordinates carried over to the next stage are $x_{k-1}^{\left(i\right)}=\mathrm{mod}\left(x_{k}^{\left(i\right)},\nu^{k-1}\right)$; *i *= 1,2,3. If an equivalent coordinate of any stage does not match the list of accessible sites, the site under consideration is blocked, only those sites surviving up to stage 1 are accessible. If the point corresponds to a blocked site, at any stage of the process, it is inaccessible. This procedure of coarse graining the grid corresponds to a renormalization of the lattice. The steps discussed generate an ordinary Brownian walk on a fractal support using a constant time step. In a continuous time random walk (CTRW), the molecule has to wait for a time *t* on each site of the fractal before performing the next step. This waiting time is a random variable independently chosen at each new step according to a continuous distribution *ψ*(*t*). In our case, it is a stable Levy distribution. If in addition to the walk on a fractal support we vary the time step based on a waiting time distribution, in our simulations an inverse gamma distribution, we generate a CTRW on a fractal support. For more details on the simulation of CTRW we refer to [11].

## 4 RESULTS AND DISCUSSION

The amount of irregularity in molecule trajectories of dynamic systems of living cells or body fluids like blood and its components can be quantified in various ways. From a mathematical point of view, the anomalous exponents $\tilde{\gamma }$ measure the dependence of the future behavior on small changes in the systems' initial conditions. When the dynamic behavior is independent of the initial conditions, the associated single molecule trajectories are ergodic [6]. The single molecule trajectories are said to be non-ergodic when the dynamics depend on the initial conditions. 

Let us assume that we have measurement data for the mean square displacement (*MSD*) based on time averages extracted from non-ergodic systems. Then, we can ask the following question. How many of these infinite tracks are needed to get the same scaling exponent resulting from the sample average of an ergodic system. The practical importance of this question is related to the experiments carried out with living cells to distinguish ergodic from non-ergodic behavior. Our results show that a selection of a few non-ergodic tracks allows us to represent in the mean of non-ergodic measurements the same scaling behavior as in ergodic systems. This means that in real experiments it becomes evidently very difficult to distinguish ergodicity from broken ergodicity from a practical point of view. To formulate the problem precisely let us denote the set of data by

(10) Sγ∼ℓti,fti = Sγ∼ℓ = ti,fℓtiti∈a,b with ℓ = 1,2,3,... ,

where *a* and *b* are the lower and upper bounds of the temporal measurement interval. If this data set represents measurement points for anomalous, subdiffusive processes we know that the *MSD* based on a temporal average is given by

(11) MSDT = fℓt = Γγ∼ℓ⋅tγ∼ℓ with ℓ = 1,2,3,... ,

where *ℓ* counts the single-molecule tracks. Assuming that the number of single-molecule tracks *ℓ* out of an infinite set of possible outcomes in a non-ergodic system can be ordered as γ∼ℓ = γ∼min+Δγ∼⋅ℓ with Δγ∼ = γ∼max−γ∼min/ℓmax, we introduce the following averaging over non-ergodic single-molecule tracks *ℓ*. The averaging is carried out by minimizing the variation between the sum of the temporal averaged *MSD* data with respect to the logarithmic scaling behavior of the subpopulation. This yields the least possible variation over non-ergodic single-molecule tracks *ℓ*

(12) $$\min\left({\left|\log\left(\frac{1}{\ell_{\max}}\sum_{\ell =1}^{\ell_{\max}}f_{\ell }\left(t_{i}\right)\right)-\left({\langle \tilde{\gamma }\rangle }_{\mathrm{sub}}t_{i}+c_{\mathrm{sub}}\right)\right|}^{2}\right)\Rightarrow \left({\langle \tilde{\gamma }\rangle }_{\mathrm{sub}},c_{\mathrm{sub}}\right)\cdot$$

Eqn. (12) represents nothing more than a minimization of the squares of the errors, e.g. measurement errors. Meaning that we are deriving the characteristics of the sub-population ${\langle \tilde{\gamma }\rangle }_{\mathrm{sub}}$ and *c_sub_* by an minimization of the mean square deviation of the simulated data and the predicted model in a logarithmic representation. ${\langle \tilde{\gamma }\rangle }_{\mathrm{sub}}$ represents the “averaged” scaling exponent, while *c_sub_* is related to the pre-factor of the scaling law. To achieve the agreement between the sample *MSD*, $\tilde{\gamma }$ and the average defined in Eqn. (12), we have in addition to minimize the variation between the experimental scaling exponent $\tilde{\gamma }$ and the sub-population scaling exponent ${\langle \tilde{\gamma }\rangle }_{\mathrm{sub}}$

(13) minγ∼−γ∼subγ∼min≤γ∼sub≤γ∼max with ℓ∈N

This way of minimizing the variations allows us not only to derive bounds for the scaling exponents ${\langle \tilde{\gamma }\rangle }_{\mathrm{sub}}$ in the subpopulation of single molecules as ${\langle \tilde{\gamma }\rangle }_{\mathrm{sub}}\in \left[{\tilde{\gamma }}_{\min},{\tilde{\gamma }}_{\max}\right]$ but moreover to fix the number of single-molecule tracks in the subpopulation as $N_{\ell_{\max}}$. It turns out that the optimal number of tracks $N_{\ell_{\max}}$ is a small number taken from an infinite set of possible values $\tilde{\gamma }$. In Fig. (**1**), the global minima occurring in this minimization process of single-molecule variations are shown for the simulated scaling exponent $\tilde{\gamma }$ = 0.689. The bounds for $\tilde{\gamma }$ are given in general by Eqs. (**12**) and (**13**). Fig. (**1**) shows how the simulated scaling behavior of the exponent $\tilde{\gamma }$ changes in a subpopulation of simulated single-molecule variations. The curves in Fig. (**1**) represent the variation of the simulated scaling exponent compared with the sub-population exponent ${\langle \tilde{\gamma }\rangle }_{\mathrm{sub}}$ if the number of simulated tracks included in the optimization (12) changes. The different curves are related to the maximal scaling exponent ${\tilde{\gamma }}_{\max}$ taken from the set ${\tilde{\gamma }}_{\max}=\left\{0.785,0.795,0.797,0.799.0.8\right\}$. These values are a result of the simulations published in ref. [11]. The values ${\tilde{\gamma }}_{\max}$ are assigned to the curves from top to bottom and, respectively, they are selected at the far right side of the Fig. (**1**) from top to bottom.

The global minima of ${\langle \tilde{\gamma }\rangle }_{\mathrm{sub}}$ are determined under the constraint of Eqn. (13), i.e. ${\tilde{\gamma }}_{\min}\leq {\langle \tilde{\gamma }\rangle }_{\mathrm{sub}}\leq {\tilde{\gamma }}_{\max}\wedge \ell \in \mathbb{N}$. The minimization of $\left|\tilde{\gamma }-{\langle \tilde{\gamma }\rangle }_{\mathrm{sub}}\right|$ is carried out under the constraint that the lower and upper boundary of the $\tilde{\gamma }$-values are unknown. These variations of the interval $\left[{\tilde{\gamma }}_{\min},{\tilde{\gamma }}_{\max}\right]$ are determined by the minimization of Eqn. (13). The resulting intervals of the minimization of Eqn. (13) under the change of $\tilde{\gamma }$ itself are shown in Fig. (**2**). The global minima of the variations are determined by $\tilde{\gamma }$ as shown in Fig. (**2**). We depict an example of averaging and the resulting scaling behavior of the $\tilde{\gamma }$-interval $\left[{\tilde{\gamma }}_{\min},{\tilde{\gamma }}_{\max}\right]$ in subpopulations of biomacromolecules for the chosen simulated value $\tilde{\gamma }$ = 0.689 of Eqn. (5). Boundary values $\left[{\tilde{\gamma }}_{\min},{\tilde{\gamma }}_{\max}\right]$ of dynamically distinct regions change with different simulated $\tilde{\gamma }$ values. The $\tilde{\gamma }$-interval $\left[{\tilde{\gamma }}_{\min},{\tilde{\gamma }}_{\max}\right]$ for single-molecule tracks in a subpopulation shows a certain bandwidth. Only within that bandwidth, variations of single-molecule responses are possible. Thus, we are able to characterize the response pattern of single-molecule variations by the heterogeneity parameter *γ*, which is modulated by the network of interactions. *γ* is experimentally accessible by means of fluorescence correlations spectroscopy and two-color fluorescence crosscorrelation spectroscopy. The dynamic interaction and cellular function of a cellular protein is modulated by up to 100 different proteins at different sites in the cell [18-24]. The spatio-temporal organization is achieved by cellular networks that we characterize by their temporal heterogeneity *γ*.

Fig. (**3**) shows an ensemble of 32 randomly selected single-molecule tracks represented as dots. These tracks are the basis of the determination of ${\langle \tilde{\gamma }\rangle }_{\mathrm{sub}}$ based on the minimization processes (12) and (13). Each of these tracks shows a specific scaling exponent which is combined in the minimization to a common scaling exponent. The most striking feature of performing ensemble averaging in sparse subpopulations of single molecules, however, is a mean value ${\langle \tilde{\gamma }\rangle }_{\mathrm{sub}}$ of the solid green line in Fig. (**3**) that is the same mean value obtained in an ergodic system. Hence, broken ergodicity and unbroken ergodicity are not anymore distinguishable. In addition, when averaging procedures are carried out withoutknowing whether the underlying molecular system behaves in ergodic or non-ergodic ways, each measurement can be related to an ergodic or a non-ergodic behavior unless one is able to show the single-molecule fingerprint of non-ergodicity.

How does the merging of variations of single-molecule tracks by ensemble averaging in a sparse subpopulation under broken ergodicity affect the primary observable in fluorescence fluctuation spectroscopy and imaging that is ‘fluorescence fluctuations’? Changes in fluorescence intensity reflect the time-averaged molecule number fluctuations of a molecular system. Here, we record the absolute number of molecule events *X* occurring in a period of *T* units time (infinite number of periods of *T* units time). The events x = 1 molecule, x = 2 molecules, etc. happen with an average detection probability *P* per unit time. For example, we record the number of molecules passing ΔV in each of 200 different 10-microsecond periods. The theoretical frequency was first derived thus [36]

(14) $$P\left(X\leq x,P\cdot T=C\right)=\int_{0}^{T}{P\cdot e}^{-P\cdot t}\cdot \frac{1}{\left(x-1\right)!}\cdot P^{x-1}\cdot {\left(T-t\right)}^{x-1}\cdot e^{-P\cdot \left(T-t\right)}\mathrm{dt}=\frac{{\left(P\cdot T\right)}^{x}}{x!}\cdot e^{-P\cdot T}=\frac{C^{x}\cdot e^{-C}}{x!}\cdot$$

The mean number of molecules in ΔV observed per period time is given by the total of molecules observed in a total of different time periods. Hence, the mean value of x happenings in the observation volume ΔV recorded or taken over an infinite number of time periods denotes the mean value of the subpopulation of molecules and equals* P·T *= *C*. By this representation Eqn. (14), we have immediate access to the measurable value of $C=c_{m}\cdot N_{A}\cdot \Delta V$, where the molar concentration of other molecules of the same kind in the bulk is *c_m_* and Avogadro's number of [mol^-1^] is *N_A_*. In order to guarantee that the Poisson probability ln$\left\{N\right\}\equiv \ln\left\{P\left(X=1,C\right)\right\}=\ln\left\{P_{1}\right\}=\ln C-C$ (see Eqn. (14)) of finding a single molecule in the femtoliter-sized observation volume ΔV= 0.14·10^-15^ [L] is *N* < 1 [37], a cut-off at about 11 nM bulk concentration is motivated for ΔV = 0.14 ·10^-15^ [L]. For *C* << *e^-C^*, it follows straightforwardly $N\equiv P_{1}≅C$ [37]. If the random waiting time distributions *ψ*(*t*) are sampled at points which do not fulfill the condition *N* < 1, we must be aware of aliasing effects with terms of two, three, etc. molecules at the same time in the observation volume ΔV = 0.14·10^-15^ [L]. 

Let us ask now how long does it take to record 32 different single molecules of the same kind in the observation volume ΔV = 0.14·10^-15^ [L]? The probability that the entering molecule is the original molecule was found to be $p_{\bar{n,n}}=1-p_{n,n}=N$, where *p_n,n_* is the reentry probability for non-meaningful reentries [14]; if the selfsame molecule does not diffuse out or in the observation volume ΔV then there is a non-meaningful molecular situation and, therefore, no temporal fluctuations in the fluorescence intensity traces of that molecule. Hence, the meaningful time *T_m_* to observe just one single molecule in ΔV is [14]

(15) $$T_{m}=\frac{\tau_{D}}{c_{m}\cdot N_{A}\cdot \Delta V\cdot \exp\left\{-c_{m}\cdot N_{A}\cdot \Delta V\right\}}\cdot$$

τ_*D*_ is the diffusion time of the molecule. This is the exact physical solution for the time that one can study the same molecule within ΔV [14].The probability that the single observation has the given variate value *X* = 1 is the proportion of times the variate-value *X* = 1 turns up when a larger number (theoretically, infinity) of random selections are made. The proportion of times that the same single molecule turns up exactly equals the proportion of individual molecules in the subpopulation which have a variate-value *X *= 1, in the long run *T*. Hence, given a subpopulation of different single molecules of the same kind, the ratio *T/T_m_* that a randomly selected molecule has a variate-value *X* = 1 allows to compare different individual molecules $N_{\ell_{\max}}$ of the same kind in the subpopulation at the measured time-averaged molecule number *N* < 1 per observation volume ΔV

(16) $$N_{\ell_{\max}}=\frac{T}{T_{m}}\cdot$$

We can, each time *T_m_* we take a sample, calculate the number of different single molecules of the same kind $N_{\ell_{\max}}$ = 32: which are recorded or taken over a finite longer time period *T, *by the useful and simple Eqn. (16). We found that for any choice at all for *N* < 1 there is a solution whose probability is given by the second and third criterion: the analytical sensitivity to detect a single molecule [37] and the arrival and departure probability of the same single molecule [15, 38] (see also Fig. 48.2 in ref. [39]: Synopsis of a new physically grounded technology of fluorescence fluctuation spectroscopy for observing single molecules at longer time scales than currently available). The corresponding discussion of Baumann and Földes-Papp [17] also applies here.

$\tilde{\gamma }$ is related by Eqs. (8)-(10) to anomalous diffusive motion in fluorescence correlation spectroscopy and two-color fluorescence crosscorrelation spectroscopy. As indicated in the method section, *D_app_*(*t*) is a function of the continuously but slowly varying network conditions $\tilde{\gamma },i.e.\tilde{\gamma }=f\left(\gamma \right)$. We assume that the distribution of exposure to interactions depends on the square root of the mean square displacement. The longer the diffusive path of the molecules, the broader the distribution of exposure to ligands (biochemical traps). We mean that the molecules are more frequently exposed to neighboring ligands, the longer their diffusive paths become. This is a very reasonable assumption. The property of self-similarity implies that a scaling relation exists between the structure observed at one scale *r* and that found at subsequent scales. However, this is not the scaling that assumes γ∼ = α⋅γ with 0<α,γ∼<1 is uniform but rather a new kind of scaling that is filled with heterogeneity*γ*. Due to different selectivity in the biological binding events of a crowded and highly heterogeneous environment like a living cell or body fluids like blood and its components, we choose the so-called Weibull distribution as the frequency distribution of exposure to interactions for molecules.

(17) $$\frac{m\left(<r\right)}{m_{\mathrm{total}}}=1-\exp\left\{-{\left(\frac{r}{r_{\max}}\right)}^{q}\right\},$$

where *m*(< *r*) is the cumulative exposure of molecule species with size less than *r*, *m_total_* is the total exposure of molecule species, and *r_max_* is related to their maximum size given by the geometrical size of the living cell or its nucleus (geometrical dimension). The power *q* is an arbitrary constant but is taken to be a positive integer; *q* is the parameter of the distribution. Because molecules have different shapes it is convenient to take a linear dimension *r* as the cube root of the molecule volume $r\propto {℧}^{-1/3}$. For the total exposure to molecular complexes and species, respectively, we can write

(18) $$m_{\mathrm{total}}=m\left(>r\right)+m\left(<r\right),$$

where* m*(*>r*) is the cumulative exposure of molecular complexes with size greater than *r*. Hence, expansion according to McLaurin's formula yields

(19) $$\frac{m\left(>r\right)}{m_{\mathrm{total}}}=\exp\left\{-{\left(\frac{r}{r_{\max}}\right)}^{q}\right\}=\sum_{h=0}^{\infty }{\left(-1\right)}^{h}.\frac{{\left(\frac{r}{r_{\max}}\right)}^{q\cdot h}}{h!}\equiv 1-{\left(\frac{r}{r_{\max}}\right)}^{q}\pm ...$$

in which higher powers of ${\left({r/r}_{\max}\right)}^{q\cdot h}$ are neglected. By substitution (19) into the exposure-frequency distribution (17), we obtain

(20) $$\frac{m\left(<r\right)}{m_{\mathrm{total}}}={\left(\frac{r}{r_{\max}}\right)}^{q}\cdot$$

Eqn. (20) reduces the Weibull distribution (17) to a power law for small *r*. The power-law scaling (20) describes how the property *m*(<*r*)/*m_total_* of molecular exposure to interactions sites (complexes) depends on the scale *r* at which it is measured. We now turn to Eqs. (17) and (20). It is often convenient to specify a distribution with a probability density function (PDF). Taking the derivative of the Weibull relationship (17), we obtain the density function *f_Weibull_*(*r*), which is the Weibull function, and apply Eqn. (19) to it

(21) fWeibull r = q⋅rq−1rmaxq⋅exp−rrmaxq = q⋅rq−1rmaxq−r2⋅q−1rmax2⋅q⋅

In our Eqn. (21), *f_Weibull_*(*r*)d*r* is the fraction of interactions with size between *r* and *r*+d*r*. The integral of Eqn. (21) from *r* = 0 to *r* = ∞ is unity because it includes all molecule interaction sites. The probability density (PDF) for the power-law distribution (20) is

(22) $$f_{\mathrm{power}-\mathrm{law}}\left(r\right)=q\cdot \frac{r^{q-1}}{r_{\max}^{q}}\cdot$$

Assuming *q* > 0 , the average interaction size is determined by the first moment of Eqn. (22) that is

(23) $$\langle r\rangle =\frac{q}{q+1}\cdot r_{\max}\cdot$$

The variance about this average interaction size $\langle r\rangle$ is

(24) $$\sigma_{\langle r\rangle }^{2}=\frac{q}{\left(q+2\right)\cdot {\left(q+1\right)}^{2}}\cdot r_{\max}^{2}\cdot$$

In Fig. (**4**), the measured *r*_max_ value of the nucleus of a HeLa cells is 22.8 µm. Taking the scale exponent *q* = 2 in Eqn. (21), we obtain a quadratic Weibull distribution of exposure to interaction sites with a mean interaction size $\langle r\rangle$ = 15.2 µm and $\sigma_{\langle r\rangle }^{2}$ 28.88 µm^2^ (Eqs. (23) and (24)). Thus, the upper limiting value of the *MSD* is $\langle {\vec{r}}^{2}\rangle$ = 2.31·10^-10^± 2.92·10^-11^ [m^2^].

In summary, the probability to perform *n* steps during time *t* is denoted by *χ_n_*(*t*), which is related to the waiting time distribution by the Laplace transform

(25) $$L\left(\chi \left(t\right)\right)=\chi_{n}\left(s\right)={\left(\psi \left(s\right)\right)}^{n}\cdot \left(1-\psi \left(s\right)\right)/s\cdot$$

This probability is needed to analyze the *MSD* for a random walk on a fractal support carried out as a CTRW [41, 42]. The probability *χ_n_*(*t*) of the random waiting time distributions *ψ*(*t*) is sampled at points which fulfill the condition *N* < 1 per observation volume ΔV and Eqn. (16). The *MSD* traveled by the molecule during the time *t* is given by

(26) $$\langle {\vec{r}}^{2}\left(t\right)\rangle =\sum_{n=0}^{\infty }\langle {\vec{r}}_{n}^{2}\rangle \cdot \chi_{n}\left(t\right),$$

where $\langle {\vec{r}}_{n}^{2}\rangle$ is the average distance traveled in *n* steps on the fractal. $\langle {\vec{r}}_{n}^{2}\rangle$ is determined by the experimentally accessible condition of Eqn. (23). In our experiment Fig. (**4**), $\langle {\vec{r}}_{n}^{2}\rangle$= 2.31·10^-10^± 2.92·10^-11^ [m^2^]. Here, we first introduce this kind of CTRW, which we call Limited Continuous Time Random Walks (LCTRW) on fractal supports.

## CONCLUSIONS

Anomalous diffusion behavior is an important issue especially in cellular single-molecule measurements and ways to precisely quantify this behavior are in high demand. Here, we present an approach on how to decide from a subset of single-molecule measurements how heterogeneous the studied system is in time. Specifically, we present an approach to distinguish between ergodic and non-ergodic behavior. We have proposed a change of the molecular behavior when single molecules are trapped in interactions with their neighboring ligands and reaction partner(s), respectively or/and by conformational changes in a crowded environment. We assume that spatial and temporal conditions are decoupled. *α* is the spatial, molecular crowding parameter and *γ* is the heterogeneous parameter of the temporal randomness. In this original research article, we present solutions to the problem how bulk egodicity behaves for subpopulations of biomacromolecules and in what ways, and by how much the interaction network of single molecules can be rendered non-ergodic by ensemble averaging during the measurement. We display the notations, introduce our definitions and report some general results. The complete absence of spatial geometry *α* is, of course, the simplest assumption [43] but more complicated structures have been considered in our models by numerical simulation on fractal supports. Different physical situations correspond to different values of the experimentally accessible parameters α⋅γ = γ∼ with 0<α,γ∼<1. The novel theory presented here offers a new way to understand the molecular behavior when single biomacromolecules are trapped in interactions with their neighboring ligands and reaction partner(s), respectively, in a crowded environment.

## Figures and Tables

**Fig. (1):**
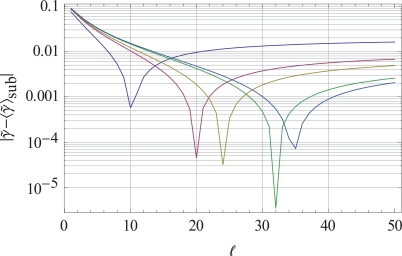
The minima of the variations $\left|\tilde{\gamma }-{\langle \tilde{\gamma }\rangle }_{\mathrm{sub}}\right|$ are shown as a function of the number of single molecule tracks $N_{\ell }\in \mathbb{N}$. In these graphs, the lower bound ${\tilde{\gamma }}_{\min}$ is fixed to the value ${\tilde{\gamma }}_{\min}$ =0.243 representing the optimal choice for the experimental $\tilde{\gamma }$= 0.689. The curves are assigned to the upper bounds of $\tilde{\gamma }$ from top to bottom. On the right side of the figure we have ${\tilde{\gamma }}_{\max}=\left\{0.785,0.795,0.797,0.799.0.8\right\}$. We identify that for a given $\tilde{\gamma }$= 0.689 the interval for selecting the random number of single-molecule tracks from the total ensemble of singlemolecule macromolecules in the subpopulation (the total ensemble of single-molecule tracks) should range from $\left[{\tilde{\gamma }}_{\min},{\tilde{\gamma }}_{\max}\right]$ = [0.243, 0.799]; for this range ${\langle \tilde{\gamma }\rangle }_{\mathrm{sub}}\in \left[{\tilde{\gamma }}_{\min},{\tilde{\gamma }}_{\max}\right]$, we find the minimal variation if the number of randomly selected single-molecule tracks is $N_{\ell_{\max}}=32$. All other values of $N_{\ell }$ deliver only a local minimum instead of a global minimum. The graphs also show that the variation approaches a stable value if $N_{\ell }$ approaches large values; i.e. only a small subpopulation of single molecules delivers the minimal variation.

**Fig. (2):**
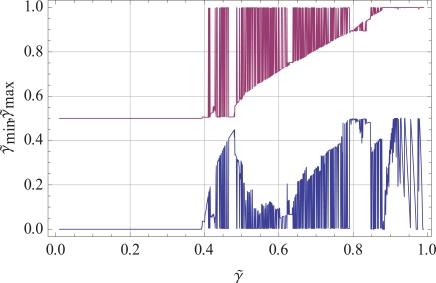
The variation of the $\tilde{\gamma }$-interval $\left[{\tilde{\gamma }}_{\min},{\tilde{\gamma }}_{\max}\right]$ with respect to the MSD scaling behavior $\tilde{\gamma }$ in the optimization process for deriving sub ${\langle \tilde{\gamma }\rangle }_{\mathrm{sub}}$ by using Eqs. (12) and (13). In the graph we can distinguish different domains for$\tilde{\gamma }$. The first domain ranges from 0 < $\tilde{\gamma }$ < 0.392… allowing a fixed interval for the limits $\left[{\tilde{\gamma }}_{\min},{\tilde{\gamma }}_{\max}\right]$ = [0, 1/2]. For values 0.392…< $\tilde{\gamma }$ < 0.878…, we observe a highly structured set of intervals where the upper and lower limit reaches some minimal or maximal value and allows the whole range [0, 1] for specific values. The optimal number of tracks is for both $\tilde{\gamma }$-intervals equal to 32. For the last interval 0.878…< $\tilde{\gamma }$ < 1, we observe the maximal value ${\tilde{\gamma }}_{\max}\approx 1$ while the lower limit ${\tilde{\gamma }}_{\min}$ varies between 0 < ${\tilde{\gamma }}_{\min}$ <1/2 . For this last domain of $\tilde{\gamma }$, the number of tracks decreases to a smaller value $N_{\ell_{\max}}$ < 32. The resolution in $\tilde{\gamma }$ to derive the shown plot was Δ$\tilde{\gamma }$ =0.00078125.

**Fig. (3):**
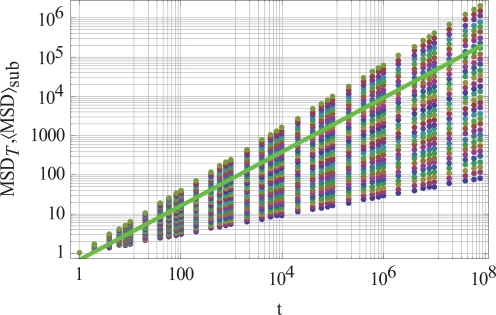
The graph shows a random selection of $N_{\ell_{\max}}$ = 32 single-molecule tracks (dots) within the bounds $\left[{\tilde{\gamma }}_{\min},{\tilde{\gamma }}_{\max}\right]$ determined in the optimization for a given value of $\tilde{\gamma }$= 0.689. The graph is using the data listed in Fig. 1. The solid green line corresponds to the average sub ${\langle \tilde{\gamma }\rangle }_{\mathrm{sub}}$ over the 32 randomly selected single-molecule tracks.

**Fig. (4):**
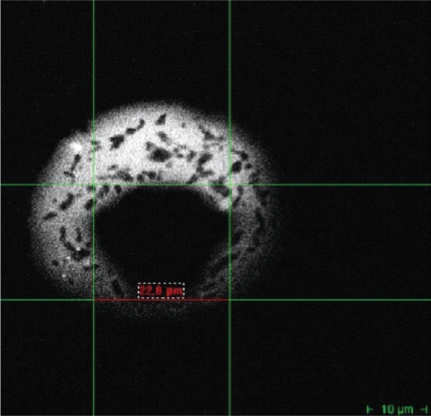
A single HeLa cell was optically sectioned by two-photon imaging after transfection with an Alexa488-labeled short RNA duplex (SQ-dsCon2) in order to visualize the geometrical dimension of the cell nucleus, i.e. its measured geometrical size. Two-photon imaging is described elsewhere [40].
